# Thiosemicarbazide Derivatives Decrease the ATPase Activity of *Staphylococcus aureus* Topoisomerase IV, Inhibit Mycobacterial Growth, and Affect Replication in *Mycobacterium smegmatis*

**DOI:** 10.3390/ijms22083881

**Published:** 2021-04-09

**Authors:** Aleksandra Kowalczyk, Agata Paneth, Damian Trojanowski, Piotr Paneth, Jolanta Zakrzewska-Czerwińska, Paweł Stączek

**Affiliations:** 1 Department of Molecular Microbiology, Faculty of Biology and Environmental Protection, University of Łódź, Banacha 12/16, 90-237 Łódź, Poland; aleksandra.strzelczyk@biol.uni.lodz.pl; 2 Department of Organic Chemistry, Faculty of Pharmacy, Medical University of Lublin, Chodźki 4a, 20-093 Lublin, Poland; 3 Department of Molecular Microbiology, Faculty of Biotechnology, University of Wrocław, Joliot-Curie 14a, 50-383 Wrocław, Poland; dtrojanowski.box@gmail.com (D.T.); jolanta.zakrzewska@uni.wroc.pl (J.Z.-C.); 4 Institute of Applied Radiation Chemistry, Łódź University of Technology, Żeromskiego 116, 90-924 Łódź, Poland; piotr.paneth@p.lodz.pl; 5 International Centre for Research on Innovative Biobased Materials (ICRI-BioM)—International Research Agenda, Łódź University of Technology, Żeromskiego 116, 90-924 Łódź, Poland

**Keywords:** thiosemicarbazides, antibacterial agents, molecular modelling, bacterial type IIA topoisomerases, DNA replication, time-lapse microfluidic microscopy

## Abstract

Compounds targeting bacterial topoisomerases are of interest for the development of antibacterial agents. Our previous studies culminated in the synthesis and characterization of small-molecular weight thiosemicarbazides as the initial prototypes of a novel class of gyrase and topoisomerase IV inhibitors. To expand these findings with further details on the mode of action of the most potent compounds, enzymatic studies combined with a molecular docking approach were carried out, the results of which are presented herein. The biochemical assay for 1-(indol-2-oyl)-4-(4-nitrophenyl) thiosemicarbazide (**4**) and 4-benzoyl-1-(indol-2-oyl) thiosemicarbazide (**7**), showing strong inhibitory activity against *Staphylococcus aureus* topoisomerase IV, confirmed that these compounds reduce the ability of the ParE subunit to hydrolyze ATP rather than act by stabilizing the cleavage complex. Compound **7** showed better antibacterial activity than compound **4** against clinical strains of *S. aureus* and representatives of the *Mycobacterium* genus. In vivo studies using time-lapse microfluidic microscopy, which allowed for the monitoring of fluorescently labelled replisomes, revealed that compound **7** caused an extension of the replication process duration in *Mycobacterium smegmatis*, as well as the growth arrest of bacterial cells. Despite some similarities to the mechanism of action of novobiocin, these compounds show additional, unique properties, and can thus be considered a novel group of inhibitors of the ATPase activity of bacterial type IIA topoisomerases.

## 1. Introduction

The need for novel antibacterial agents to effectively treat drug-resistant infections remains unfulfilled [1,2,3,4,5]. The accumulation of mutations in DNA gyrase and topoisomerase IV genes, jeopardizing the efficacy of fluoroquinolones [6], is an example of such resistance development and highlights the urgent need to develop novel antibiotic scaffolds. Even though resistance to fluoroquinolones among clinical isolates was detected relatively quickly after their introduction [7], bacterial topoisomerases still remain clinically validated targets due to their conservation across all bacteria and also their strong structural differences to eukaryotic topoisomerases [8,9,10,11,12]. Moreover, because of the structural similarities between DNA gyrase and topoisomerase IV, dual targeting is possible, which prolongs the onset of resistance [13,14,15,16,17,18,19].

DNA gyrase and topoisomerase IV (Topo IV) belong to the group of DNA topoisomerases, the enzymes essential for cell viability. They are critical for maintaining the topological state of DNA by homeostatic control of global and local supercoiling, as well as introducing or removing knots and tangles in DNA molecules during processes such as DNA replication, transcription, DNA repair, and recombination [20,21]. In particular, DNA gyrase is primarily responsible for reducing the linking number of the DNA, and thus the introduction of negative supercoils into DNA, whereas Topo IV is involved in the decatenation of chromosomes after replication, DNA unknotting, and relaxation of supercoiled DNA [22,23,24,25]. Both enzymes were found to be involved in resolving the DNA topological conflicts which occur during head-on collisions between replication and transcription machinery [26].

These two enzymes are homologous heterotetrameric proteins comprised of two GyrA and two GyrB subunits, which together form an A_2_B_2_ complex for gyrase, or two ParC and two ParE subunits forming a C_2_E_2_ complex for topoisomerase IV, respectively. The GyrA and ParC subunits are involved in the DNA cutting and resealing process, while the GyrB and ParE subunits contain ATPase domains [21]. So far, two antibiotic classes, fluoroquinolones [27] and aminocoumarins [28], have clinically validated DNA topoisomerases as viable targets. The fluoroquinolones bind at the enzyme–DNA interface in the cleavage–ligation active site, where they stabilize double-stranded breaks in the bacterial DNA, induce rapid killing of the cells, and exhibit broad-spectrum antibacterial activity [29]. In turn, the aminocoumarins bind to the ATPase subunits of topoisomerases—GyrB and, to a lesser extent, ParE, and block the ATP hydrolysis function of the proteins. Aminocoumarins exhibit antibacterial activity that is mostly limited to Gram-positive pathogens [30,31]. Novobiocin is the only aminocoumarin ever licensed for clinical use. However, due to reasons of safety or effectiveness, and the introduction of more effective antibiotics, it was later withdrawn from the market. Despite the efforts to discover further GyrB or dual targeting GyrB/ParE inhibitors, no other antibiotics have advanced into the clinic so far, although a plethora of compounds, both natural and synthetic, have been shown to exhibit antibacterial activity by competing with ATP in DNA gyrase and topoisomerase IV [32,33]. In turn, widespread availability and uncontrolled use of the second class of bacterial DNA topoisomerases inhibitors, i.e., fluoroquinolones, has contributed to the rapid emergence of resistance to these agents [13,34,35]. Despite the fact that the resistance to fluoroquinolones is still increasing in numerous bacterial species, clinical isolates of fluoroquinolone-resistant bacteria are typically susceptible to ATPase inhibitors with no elevation in the minimal inhibitory concentration (MIC) [36]. Furthermore, there are no well-documented examples of bacterial resistance to ATPase inhibitors in the clinic. Thus, the search for new chemical scaffolds targeting the ATPase activity of bacterial DNA topoisomerases has emerged as an important strategy in the development of novel antibacterial agents.

**Figure 1:**
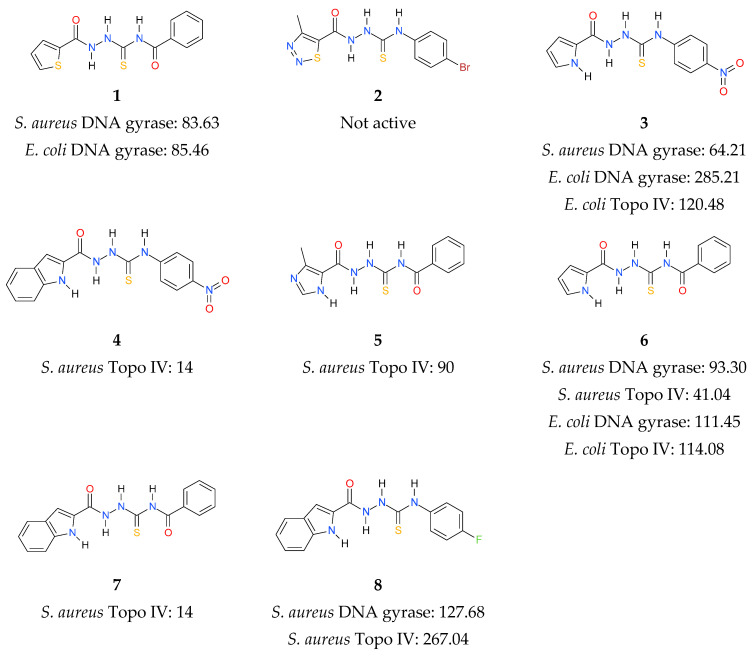
Inhibitory activity (IC_50_, (µM)) of thiosemicarbazide derivatives **1**–**8** against bacterial DNA topoisomerases [37,38,39]. Gyrases were tested for their supercoiling activity using relaxed pBR322 as a substrate, while Topo IV compounds were tested for their decatenation activity using kDNA.

In continuation of the above efforts, we have reported the synthesis of thiosemicarbazide derivatives, which act as inhibitors of bacterial topoisomerases (Figure 1) [37,38,39]. The results presented in our previous work culminated in the identification of 4-benzoyl-1-(indol-2-oyl)thiosemicarbazide (compound **7**) as the initial prototype of a novel class of bacterial DNA gyrase and Topo IV inhibitors. To expand these initial findings with more detail on the mode of action, further studies were undertaken, the results of which are presented herein.

## 2. Results and Discussion

### 2.1. Rationale

**Figure 2:**
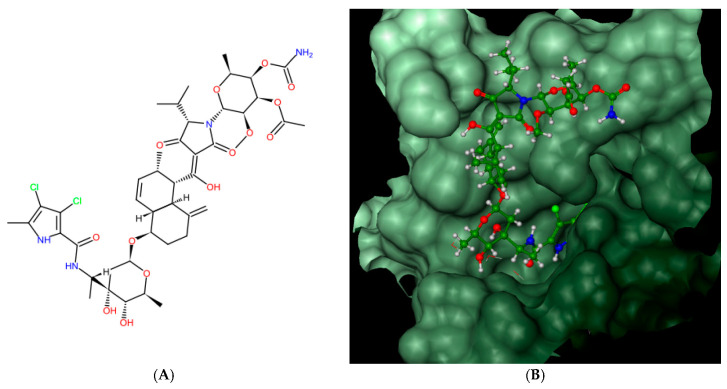

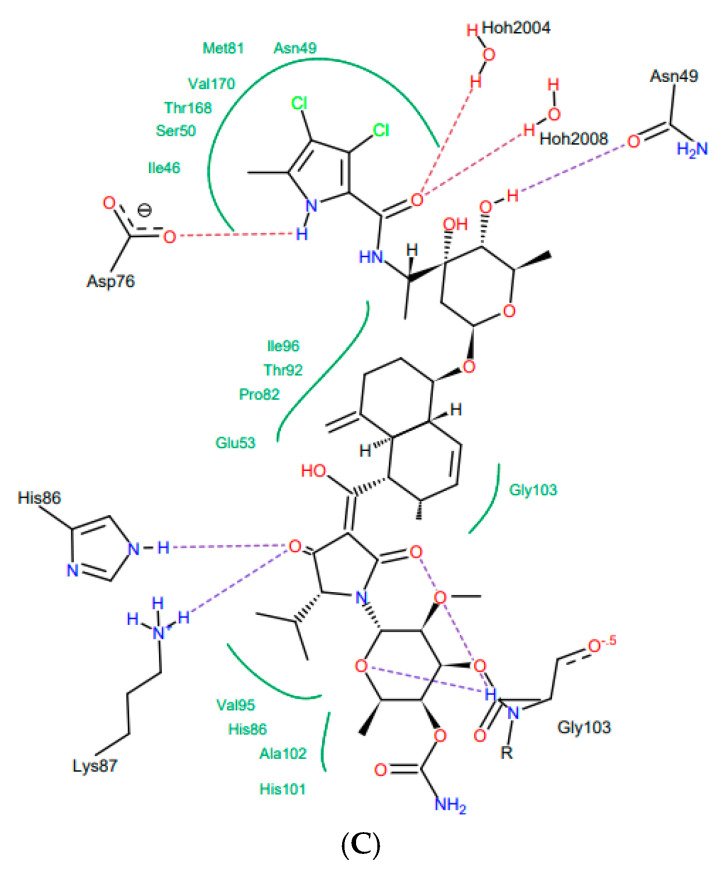
Kibdelomycin: structure (**A**), best pose in binding to *S. aureus* 43 kDa ParE (Protein Data Bank (PDB) entry: 4URL) (**B**), and interactions with amino acids in the active site (**C**).

Bacterial topoisomerases (DNA gyrase and Topo IV) belonging to class IIA utilize the energy from ATP hydrolysis to catalyze topological transactions in the DNA that are crucial for bacterial life. Gyrase, together with topoisomerase I, maintain global DNA supercoiling homeostasis and also act locally during processes requiring DNA unwinding, such as DNA replication or transcription. These processes result in the accumulation of positive supercoils ahead of the advancing enzyme or protein complex (e.g., replisome), and negative supercoils are formed behind it. The excess of both types of supercoils needs to be removed to provide proper DNA metabolism. Along with the supercoiling changes, metabolic processing of the DNA double helix results in the formation of intramolecular knots and intermolecular catenanes. While catenanes impair the resolution of the newly replicated, interlinked sister chromosomes or plasmids, knots may interrupt the movement of the enzymes, such as RNA polymerase, along the DNA [40] and weaken the strength of the DNA filament [41]. Both types of DNA tangles were proposed to be involved in the formation of the topological barriers dividing bacterial chromosomes, also referred to as the nucleoid, into superhelical domains [42]. Topo IV was found to be responsible for decatenation activity, and, at the same time, it is the strongest unknotting enzyme in *E. coli* cells [24], while gyrase prepares optimally supercoiled products for these operations [43]. During their activity, both gyrase and Topo IV, in an ATP-independent manner, create a double-stranded break in one segment of DNA (called a G-segment), forming a transient “cleavage complex”. Then, using energy derived from ATP hydrolysis, another DNA segment (T-segment) is passed through this break, followed by ATP-independent phosphodiester bond restoration. Drugs belonging to the quinolone family of antibiotics, such as nalidixic acid or ciprofloxacin, can inhibit the enzyme by stabilizing the cleavage complex before resealing a DNA break, which converts the protein into an endogenic toxin and leads to cell death. Others, such as aminocoumarin antibiotics (e.g., novobiocin, clorobiocin) compete with ATP for access to its binding site, thus impairing energy production by inhibiting ATP binding and its subsequent hydrolysis. As a result of targeting the enzymes which are essential for bacterial DNA function, which differ significantly from their eukaryotic counterparts, both groups of antibiotics are considered to be potent antimicrobials. However, due to the increasing problem of drug-resistance among bacteria, the need for the development of new classes of antimicrobial drugs, including those directed against topoisomerases, has become urgent. In 2011, kibdelomycin (KBD) (Figure 2A), a natural bactericidal agent against Gram-positive bacteria, was reported as a new class of ATPase inhibitor of DNA gyrase B (GyrB) and topoisomerase IV (ParE) subunits, without cross-resistance to other known DNA gyrase inhibitors [44]. Three years later, its crystal structures in complex with *S. aureus* GyrB and ParE proteins were published, which resolved the molecular details of its interactions with those targets [45]. KBD binds to ParE in a U-shaped “dual arm” conformation (Figure 2B), and most of its contacts with the surrounding residues are through the two stretched arms. The pyrrolamide moiety of its “lower arm” occupies the same pocket as the adenine group of ATP and is H-bonded to the conserved Asp76 (or Asp81 in GyrB), while the “upper arm”, together with the hydrophobic linker connecting these arms, protrudes from the pocket and wraps around the ATP-binding domain (Figure 2C). The overall binding mode of KBD to GyrB is very similar to that of KBD bound to ParE, with only a slight shift in the “upper arm”, while most of the hydrophobic and all polar residues surrounding the pyrrolamide moiety in the lower binding pocket are identical to those in ParE.

**Figure 3:**
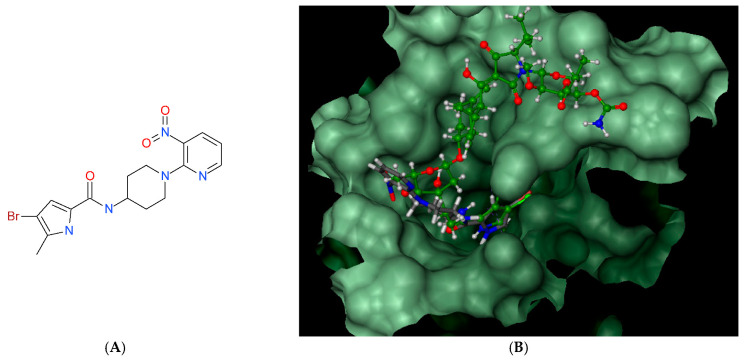

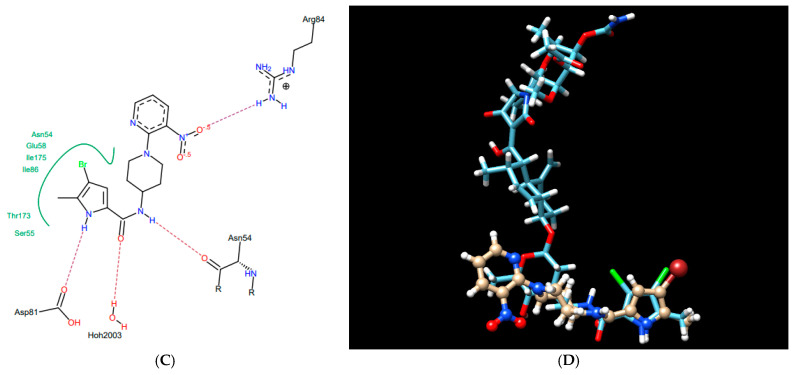
Pyrrolamide-based GyrB inhibitor: structure (**A**), best pose in binding to *S. aureus* GyrB (PDB entry: 4URM) (**B**), interactions with amino acids in the active site (**C**), and best pose overlaid with kibdelomycin (**D**).

This U-shaped binding mode of KBD is unique and distinctly different from other known GyrB/ParE small molecule inhibitors, including novobiocin, clorobiocin, and the ATP substrate analogue, ADPNP [45]. Indeed, as presented in Figure 3 for a representative small molecule pyrrolamide GyrB inhibitor [46], the binding mode of this inhibitor does not overlap with the “upper arm” of KBD, while it closely mimics the binding pose of the pyrrolamide and sugar sub-structural fragments of its “lower arm”. In a similar manner to the pyrrolamide moiety of KBD, the pyrrolamide group of the inhibitor is buried in the ATP-binding pocket and hydrogen-bound to the conserved Asp81, whereas its nitropyridine substituent extends outside of the pocket and is positioned close to the residues Arg84 and Arg54.

Other pyrrolamide and indolamide bacterial DNA topoisomerases inhibitors, such as the natural product clorobiocin [47] and synthetic compounds [36,48,49], have also been predicted by enzymatic assays or X-ray crystallography to act as ATPase inhibitors. Thus, it was reasonable to assume that our thiosemicarbazides with pyrrolamide (**3** and **6**) and indolamide (**4**, **7**, **8**) structural motifs will also bind to the ATP binding pocket. Therefore, prior to enzymatic studies, their proposed inhibitory mechanism was validated by computational docking.

### 2.2. Docking Studies

Since the FlexX program correctly reproduced the experimentally determined crystal structure of KBD in complex with *S. aureus* ParE 43 kDa (PDB entry 4URL) and closely mimicked the crystal structure of a small inhibitor molecule, pyrrolamide, within the *S. aureus* GyrB ATP-binding domain, we found this protocol suitable for the molecular docking analysis.

**Figure 4:**
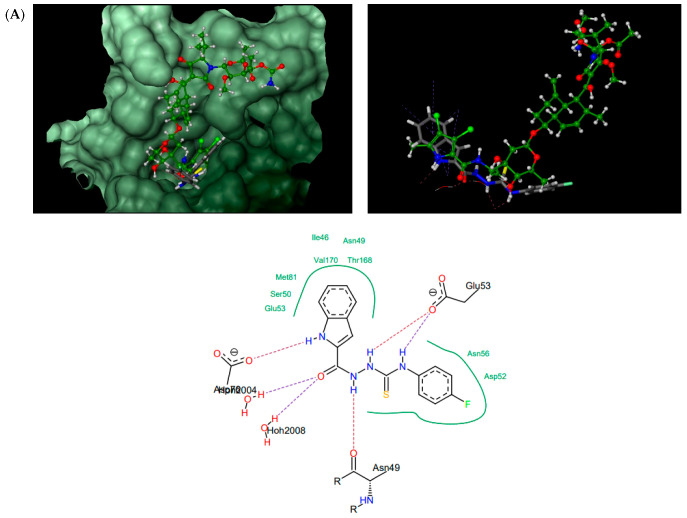

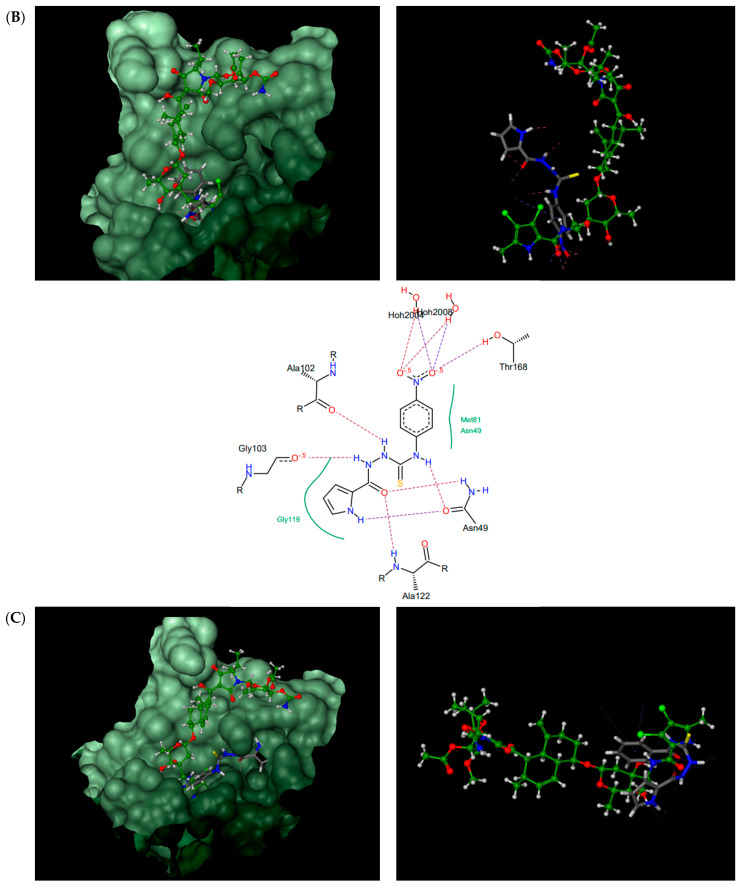

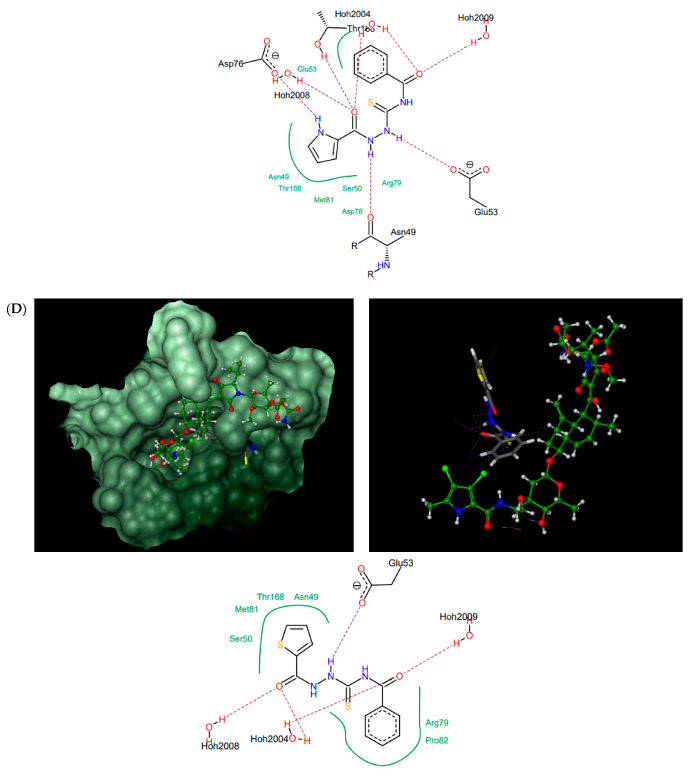
Thiosemicarbazides **8** (**A**), **3** (**B**), **6** (**C**), and **1** (**D**): best poses in binding to the ATP binding pocket of *S. aureus* 43 kDa ParE (PDB entry: 4URL) (left), best poses (in grey) overlaid with kibdelomycin (in green) (right), and interactions with amino acids in the active site (middle). With the exception of **2**, the remaining thiosemicarbazides are predicted to bind in a similar manner to **8**, both in their extended (**3, 5, 7**) or U-shaped (**4**) conformations. The binding mode of **2** is closely similar to **3**.

According to the docking results presented in Table 1, the thiosemicarbazides with an indolamide moiety (**4**, **7**, and **8**) were identified to bind to the ATP binding pocket of *S. aureus* ParE with a much higher affinity than native kibdelomycin. Their predicted binding sites overlap well with the “lower arm” of the KBD binding site, with only a slight shift of the binding site of **8**, which was out of the ATP pocket towards Asp52 (Figure 4A). In all cases, the indole nitrogen occupies an almost superimposable position with the pyrrole nitrogen of KBD and forms a hydrogen bond with conserved Asp76. The binding of the indole moiety of **4**, **7**, and **8** is further stabilized by numerous hydrophobic interactions with surrounding residues, and most of these interactions are identical to those in the crystal structure of kibdelomycin in complex with 43 kDa ParE. A hydrogen bonding network is in all cases formed between the thiosemicarbazide chain, Glu53, and water molecules, while for **4**, an additional hydrogen bond interaction between its nitro group and Ser50 is predicted. Contrary to expectations, however, the pyrrolamide core of **3** (Figure 4B) is rotated by approximately 90° relative to that in **4**, **7**, **8**, and shifted towards the sugar substructural fragment of KDB. As a result, the specific hydrogen bonding interaction between the nitrogen pyrrole of **3** and Asp 76 is lost. Instead, a rich network of intermolecular interactions involving its nitro group, residues Asn49, Ala102, Gly103, Gly119, Ala122, and Thr168, and structural water molecules is observed, which resulted in its best docking score as predicted by the docking protocol. In turn, the replacement of the indole moiety in **7** with the pyrrole led to the U-shaped **6** (Figure 4C) with a hydrogen bonding interaction with Asp76 predicted for its nitrogen atom of the pyrrole ring. The substitution of the pyrrole in U-shaped **6** with a similar in size imidazole moiety provided compound **5** in its extended binding conformation. Due to its binding similarity to the native KBD, compound **5** is expected to be a potent ATPase inhibitor as well. The imidazole nitrogen of **5** is positioned in a hydrogen bonding interaction with Asp76. In the calculated binding profiles for **1** and **2**, in turn, the binding site for **1** (Figure 4D) is close to but does not overlap with the kibdelomycin binding site, whereas the binding mode of **2** is closely similar to **3**.

**Figure 5:**
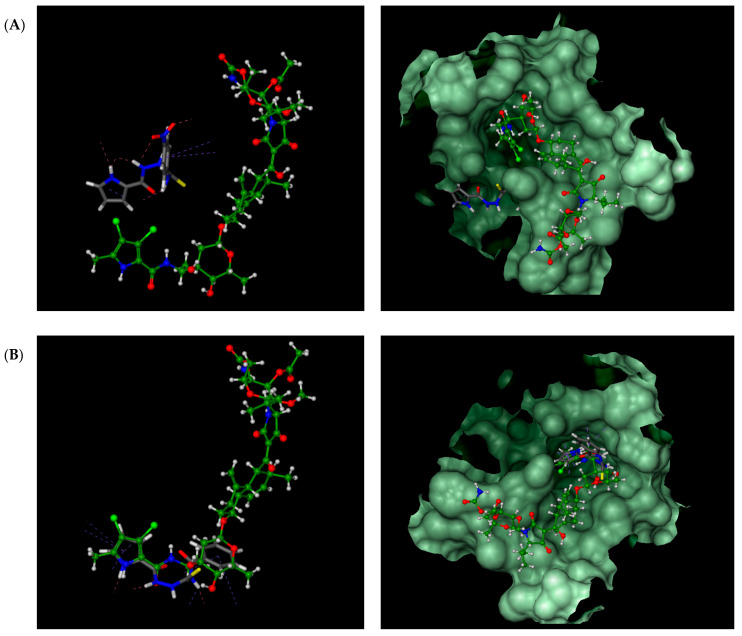
Overlay of calculated binding modes of thiosemicarbazides (in grey): (**A**) **3**; (**B**) **6** and native kibdelomycin (in green) in the ATP binding pocket of *S. aureus* GyrB (PDB entry: 4URM). Remaining thiosemicarbazides are predicted to bind in manner similar to **6** (**2**, **4**, **7**, and **9**) or to **3** (**1** and **5**), respectively.

For comparison, the binding mode of thiosemicarbazides **1**–**8** was also studied in the ATP binding site of *S. aureus* GyrB (PDB entry: 4URM). In line with expectations, all compounds are predicted to bind to the ATP binding pocket with much higher affinities than those predicted for native kibdelomycin (Table 1, second entry). As presented in Figure 5, the binding sites for **1**, **3**, and **5** are seen in a more distal region of the ATP binding pocket, while the binding mode of the remaining compounds (**2**, **4**, and **6**–**8**) is very similar to that of KBD. Hydrogen bonding interactions with the conserved Asp81 are predicted for the indole nitrogen of **4** and **7** and the pyrrole nitrogen of **6**, whereas for **8**, close hydrophobic interactions between its thiosemicarbazide chain and Asp81 are expected. Their binding to the ATP binding pocket is further stabilized by numerous hydrophobic interactions with surrounding residues, and most of these interactions are identical to those in the crystal structure of kibdelomycin in complex with GyrB.

**Figure 6:**
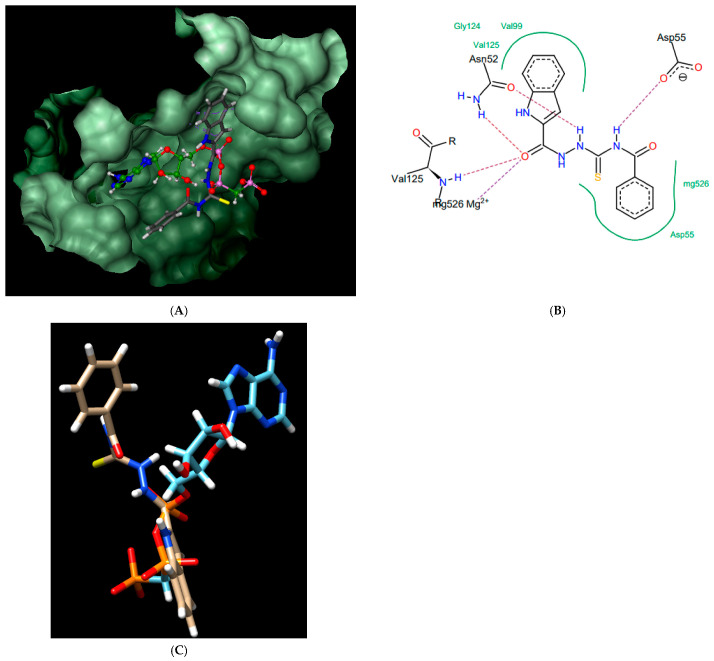
Compound **7**; best pose in binding to the ATPase region of *Mycobacterium tuberculosis* GyrB (PDB entry: 3ZM7) (**A**), interactions with amino acids in the active site (**B**), and best pose overlaid with the native inhibitor (AMPPCP) (**C**).

Compound **7**, which was later found to be the most active against two strains of the *Mycobacterium* genus, was also studied for its binding potential in the ATPase region of *Mycobacterium tuberculosis* GyrB protein (PDB entry: 3ZM7), where it was predicted to bind with a higher affinity than predicted for the native inhibitor, AMPPCP (−27.1 vs. −23.0). As presented in Figure 6, it binds to the same site as AMPPCP and is stabilized by several hydrogen bonding interactions and close hydrophobic contacts with the surrounding amino acids. However, this observation was not confirmed later in the in vitro assay against the supercoiling activity of *M. tuberculosis* gyrase (data not shown).

### 2.3. Enzymatic Assays

**Figure 7:**
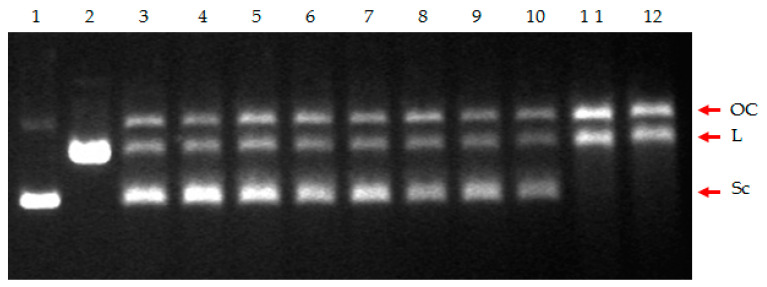
Cleavage assay of the supercoiled pBR322 plasmid DNA in the presence of *S. aureus* Topo IV and the tested compounds. Lane 1, supercoiled plasmid (control); lane 2, linearized plasmid (control); lane 3, no drug (control); lane 4, compound **4** (0.1 µM); lane 5, compound **4** (10 µM); lane 6, compound **4** (100 µM); lane 7, compound **7** (0.1 µM); lane 8, compound **7** (10 µM); lane 9, compound **7** (100 µM); lane 10, norfloxacin (0.1 µM); lane 11, norfloxacin (10 µM); lane 12, norfloxacin (100 µM). The position of nicked (OC), linear DNA (L), and supercoiled DNA (Sc) is indicated.

According to the docking studies, there was sufficient evidence that at least the inhibitors that form H-bond interactions with Asp76 (Asp81), i.e., **4**, **5**, **6**, **7**, and **8**, should exhibit inhibition of ATPase activity of type IIA topoisomerases. However, among these compounds, only **4** and **7** showed strong inhibitory activity when tested in vitro against the purified enzymes, and in both cases, it was the decatenation process conducted by topoisomerase IV that was affected the most (Figure 1). To provide experimental confirmation of the assumption that the observed inhibition of decatenation by topoisomerase IV indeed may be due to the impairment of ATPase activity and not its cleavage ability, two enzymatic tests were performed. Inhibition of the resealing process after the introduction of the double-stranded break in the DNA, proven for fluoroquinolone antibiotics, results in the accumulation of double-stranded DNA breaks and, finally, in bacterial cell death [50]. Their inhibitory action against bacterial DNA topoisomerases might be due to their interactions with DNA within the cleavage complex. Thus, in the first test, we subjected compounds **4** and **7**, as well as norfloxacin, a common drug from the fluoroquinolones group, to the non-radiolabeled cleavage assays with a supercoiled pBR322 used as the substrate. The appearance of linear DNA formed during denaturation of the drug–enzyme–DNA complex was monitored. The relaxed, linear, and supercoiled DNA forms were resolved and visualized by agarose gel electrophoresis followed by ethidium bromide staining. The inhibitory activity of norfloxacin was observed at a concentration of ≥10 µM (Figure 7, lanes 10–12). In contrast, no increase in linear plasmid DNA was detected for compounds **4** and **7** when tested at concentrations ranging from 0.1–100 µM (Figure 7, lanes 3–9).

The second assay, focused on the ability of the tested compounds to inhibit the process of ATP hydrolysis by the enzyme, confirmed the predictions of the docking studies in the case of derivatives **4** and **7** (Table 2). The percentages of the inhibition of ATPase activity of topoisomerase IV by the control drug novobiocin and the tested compounds at 20 µM concentration were similar, reaching the highest value in the case of novobiocin (67.25%) and lowered slightly in the case of **4** and **7** (55.76% and 49.35%, respectively).

Given the above results, it is clear that thiosemicarbazides **4** and **7** did not act as ParC inhibitors (topoisomerase IV subunit involved in the cleavage/resealing reaction), however, in line with the predictions arising from the molecular docking studies, they were able to reduce the rate of ATP hydrolysis by the ParE subunit similarly to novobiocin.

### 2.4. Evaluation of Antibacterial Activity of the Selected Compounds

Both compounds were tested previously against Gram-positive reference strains of *S. aureus*, *S. epidermidis*, *Micrococcus luteus*, and *Bacillus cereus* [37,38]. To expand the range of the analyzed strains, we decided to test a set of 12 clinical strains of *S. aureus* described in [51], including two MRSA strains (D15 and D17; Table 3), as well as two reference representatives of the *Mycobacterium* genus, namely *M. smegmatis* mc^2^ 155 and *M. tuberculosis* H37Rv (Table 4).

Compound **7** showed stronger growth inhibitory activity against staphylococci than compound **4** (MIC = 8–32 µg/mL vs. MIC = 64 µg/mL, respectively). For all but one strain (*S. aureus* F7), compound **7** was also 2–8 times more active than the commonly used β-lactam antibiotic ampicillin. However, two other common antibiotics (oxacillin and nitrofurantoin) were more or equally potent against all tested strains, except for *S. aureus* D15 and D17 strains (which were twice more susceptible to **7** than to oxacillin), and *S. aureus* F12 (which was twice more susceptible to **7** than to nitrofurantoin). For both thiosemicarbazide derivatives, MBC values were eight times higher than their corresponding MICs, which highlights their bacteriostatic activity. Interestingly, compound **7** showed high activity against *M. smegmatis* and *M. tuberculosis* strains, and in these cases, MIC values were equal to MBCs, indicating its bactericidal mode of action.

### 2.5. Cytotoxicity Analyses

Cytotoxic activities were preliminarily tested for compounds **1**, **3**, **5**, **6**, and **7**, as we described previously [37,38,39]. However, for a more in-depth view of the effect of compounds **4** and **7** on HeLa cells, we used the ApoToxGlo™ Triplex Assay (Promega), which enables the simultaneous measurement of cytotoxicity as well as cell viability and the induction of apoptosis. Cisplatin, a strongly cytotoxic compound with proven pro-apoptotic activity, was used as a control. HeLa cells were incubated with the tested compounds for 24 h at a concentration of 16–128 µg/mL and cisplatin at a concentration of 2–16 µg/mL.

**Figure 8:**
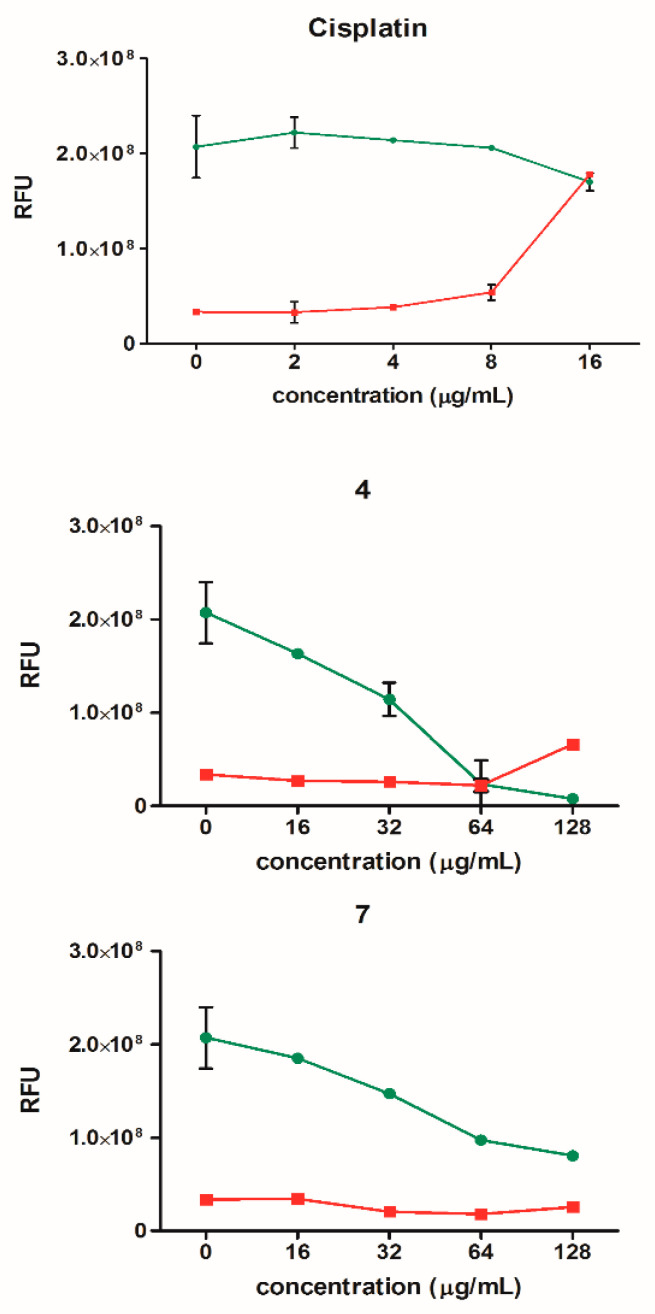
Cell viability (**green**) and cytotoxicity (**red**) of HeLa cells after treatment with compounds **4** and **7** (16–128 µg/mL) and cisplatin (2–16 µg/mL). The results are presented as mean ± SEM from two independent experiments. RFU: relative fluorescence units.

The results presented in Figure 8 show that during the treatment of the cells with the tested thiosemicarbazide derivatives, a significant decrease in the number of viable cells could be observed without any cytotoxic effect. Therefore, it can be concluded that the tested compounds probably inhibit cell proliferation, e.g., as a result of an impaired replication process, but do not cause their death. Only at concentrations above 64 µg/mL for compound **4**, a slight increase in cytotoxicity could be observed. This differed for cisplatin, where a strong increase in cytotoxicity was observed, which resulted in a decrease in cell viability. Induction of apoptosis (an increase in caspase 3/7 concentration) was seen only in the case of cisplatin (data not shown).

### 2.6. Time-Lapse Microfluidic Microscopy

**Figure 9:**
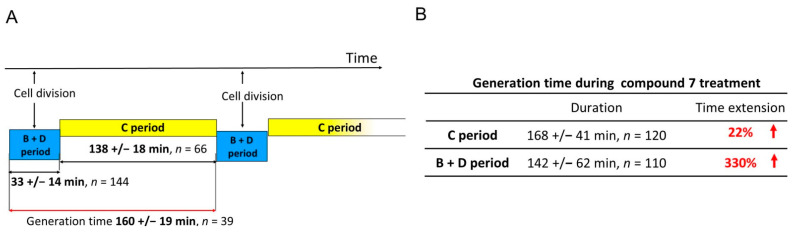
The duration of the C and the B + D periods during the cell cycle of *M. smegmatis* expressing DnaN-EGFP protein fusion (JH01 strain) measured under optimal growth conditions (**A**) and when treated with compound **7** (**B**).

Since the inhibition of the ATPase activity of type II topoisomerases may directly influence the passage of replication forks, and compound **7** revealed its high potency against *M. smegmatis*, we performed time-lapse microfluidic microscopy (TLMM) experiments using the *M. smegmatis* DnaN-EGFP (JH01) reporter strain, in which the enhanced green fluorescent protein (EGFP) encoding gene was fused to the gene encoding the sliding clamp (DnaN, beta subunit of DNA polymerase III) in its native chromosomal locus. This allowed us to observe the dynamics of the replisomes (the multiprotein complexes involved in DNA synthesis) during chromosome replication [52] and the impact of compound **7** on replisome movement at the single-cell level. The growth of this strain was similar to that of the wild-type (WT) *M. smegmatis* mc^2^ 155. In our experiments, we used a concentration of 5× the MIC of compound **7** (Table 4), because this concentration allowed us to monitor the changes in the replication dynamics without rapidly killing the bacterial cells. Mycobacterial cells were observed for 5 h under optimal conditions (without the drug), followed by 5 h of the tested compound treatment (this constituted approximately twice the chromosome replication time) and then for an additional 7 h in fresh medium after the washout of the compound. The bacterial cell cycle can be divided into the C period (the time of synthesis of daughter chromosomes, which lasts from the moment of the replication initiation until its termination), and the B + D period, which lasts from replication termination to the initiation of replication in daughter cells. In TLMM experiments, replication initiation corresponded to the appearance of a fluorescent foci of DnaN-EGFP. During the replication process (C period), the replisomes (seen as fluorescent foci) frequently split and merged back together. Finally, replication was terminated, which was observed as the disappearance of fluorescent foci (see the video in the Supplementary Materials). The duration of the C and B + D periods under optimal growth conditions and treatment with **7** is summarized in Figure 9.

**Figure 10:**
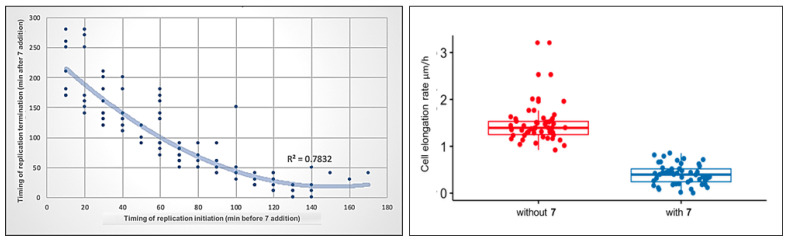
Correlation between the timing of compound **7** addition and replication initiation or termination during the cell cycle of *M. smegmatis* DnaN-EGFP (JH01) (**left**). *M. smegmatis* DnaN-EGFP (JH01) cell elongation (**right**) during treatment with compound **7** (blue dots) and before treatment during growth in optimal conditions (red dots). The difference was statistically significant with *p* = 1.4 × 10^−28^, *n* = 50.

In the presence of 4-benzoyl-1-(indol-2-oyl)thiosemicarbazide (**7**), the replisomes remained visible but their progression was decreased along the chromosome. This was observed as a moderate prolongation of the C period compared to the untreated control (mean, 168 ± 41 min, *n* = 120), which may be related to the impairment in nucleoid unknotting, resulting in the accumulation of the topological barriers defining the boundaries of superhelical domains. Two major groups of cells were observed during exposure to **7**. The first group (34% of 183 cells) comprised cells in which replisome foci were visible during the entire time of incubation with the compound due to the delay in replication fork passage. The second group comprised cells that terminated replication in the presence of **7** (66% of 183 cells). In the latter group, 50% of the cells did not initiate the next replication round in daughter cells, 8% initiated replication only in one daughter cell, and 42% initiated the next replication round in both daughter cells. Interestingly, the shorter the interval between replication initiation and the addition of compound **7**, the longer duration of replication (the C period) was observed. As shown in Figure 10 (left), the relationship is non-linear, i.e., the bacterial cells which initiated replication closer to the addition point of **7** to the medium replicated for disproportionately longer than the cells that initiated replication much earlier before the compound was added. This may suggest that the activity of the tested compound affects the process of replication initiation and/or that the inhibition of DNA untangling results in difficulties resolving sister chromosomes, which may play a role as the important checkpoint(s) before initiation of the next round of DNA replication.

Comparing the obtained results of C period duration in *M. smegmatis* cells treated with compound **7** with the results described by Trojanowski et al. [52] for known inhibitors of type IIA topoisomerases (novobiocin and nalidixic acid), it should be noted that the tested thiosemicarbazide derivative shows a mechanism of action similar to novobiocin. Novobiocin, like compound **7**, stalled the replication fork; however, the mobility of the replisomes was reduced by 175% ± 60% at 5× the IC_50_. As in the case of compound **7**, two groups of cells were observed: those in which replication lasted throughout antibiotic treatment, and those that terminated replication during the action of novobiocin. The percentage of cells in both groups was almost identical to that of compound **7**. In contrast to novobiocin and **7**, quinolones completely abolished replication.

Significant differences, however, were observed during the B + D period. In the cells terminating replication and starting a new replication round under compound **7** treatment, we observed a prominent (330%) increase in time spanning the sum of the B and D periods (mean = 142 ± 62 min, *n* = 110) compared to the untreated control. These results show that even when some of the cells complete the replication process during incubation with the tested compound, the B + D phase is significantly disturbed, which also affects the next round of replication in the daughter cells. In the case of quinolones, after disassembly of the replisomes in the presence of nalidixic acid, replication was restarted at the same sites from which they had previously disappeared [52].

The use of TLMM allows us to simultaneously observe the target process along with other processes e.g., cell growth. During exposure to compound **7**, mycobacterial cells elongated much slower (72% inhibition of the cell elongation rate) than they did in optimal conditions (Figure 10, right). Cells grew more than three times slower (mean = 0.4 µm/h, *n* = 50) compared to untreated cells (mean = 1.45 µm/h, *n* = 50). Interestingly, novobiocin had only a moderate effect on bacterial cell elongation compared to the tested thiosemicarbazide derivative: cells elongated only 30~50% more slowly than the untreated control, depending on the concentration used [52]. The similarity of the mode of action of compound **7** to novobiocin in the replication process, and, at the same time, its greater impairment of cell growth, may indicate the existence of an additional antibacterial activity of this thiosemicarbazide derivative, different from the inhibition of the ATPase activity of type II topoisomerases.

## 3. Materials and Methods

### 3.1. Docking Studies

Flexible docking was performed by means of the FlexX algorithm [53] as implemented in the LeadIT software [54]. The energies of binding of thiosemicarbazides **1**–**8** into the ATP binding pocket of *S. aureus* 43 kDa ParE (PDB entry: 4URL) and GyrB (PDB entry: 4URM), for which 3D structures were taken from the crystal structures deposited in the Protein Data Bank [55], were analyzed. The active sites were defined to include all atoms within a 6.5 Å radius of the native ligands. The first 100 top-ranked docking poses were saved for each docking run.

### 3.2. Cleavage Assay

Two thiosemicarbazide derivatives, **4** and **7**, were tested in vitro for their activity to inhibit the DNA breakage–rejoining step of the topoisomerase reaction using an *E. coli* gyrase cleavage kit, *S. aureus* gyrase cleavage kit and *S. aureus* topoisomerase IV cleavage kit (INSPIRALIS, Norwich, UK, cat no. GCK001, SAGC001, SATC001). For *E. coli* and *S. aureus* gyrases, substrate DNA (0.5 µg) of supercoiled pBR322 was incubated with 1 U of the enzyme under supercoiled conditions in 1× assay buffer in the absence of ATP. The tested compounds were added to the reaction at a final concentration of 100 µm (1% DMSO). The total volume of the sample was 30 µL. After 1 h incubation at 37 °C, the samples were treated with 3 µL of 2% SDS and 1.5 µL of 10 mg/mL Proteinase K and then incubated for 30 min at 37 °C to release trapped cleavage complexes. Then, the reaction was stopped with Stop Tris-EDTA-Bromophenol blue buffer (STEB) buffer (40% (*w*/*v*) sucrose, 100 mM Tris-HCl pH 8, 1 mM EDTA, 0.5 mg/mL bromophenol blue) and the products were analyzed by agarose gel electrophoresis (80 V, 2 h). For the *S. aureus* topoisomerase IV reaction, supercoiled pBR322 was incubated under relaxation conditions. The following steps were similar to the gyrase cleavage assay. Also, ciprofloxacin was used as a positive control drug in the gyrase assay and norfloxacin was used in the topoisomerase IV assay at concentrations of 0.1 µM, 10 µM and 100 µM. The occurrence of the linear DNA band on the agarose gel with a simultaneous decrease of the density of a band representing supercoiled DNA indicates the inhibition of enzyme activity.

### 3.3. ATPase Assay

**Scheme 1:**
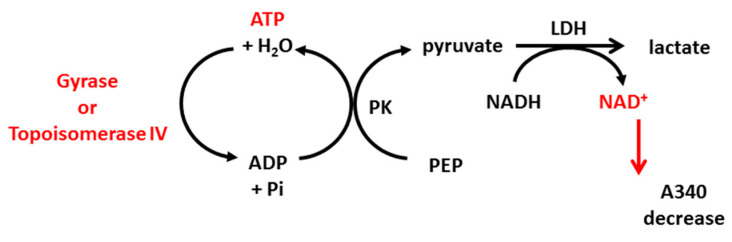
Stages of the reactions used in ATPase assay based on enzymatic ATP hydrolysis, which can be detected by absorbance decrease at 340 nm. PK: pyruvate kinase, PEP: phosphoenolpyruvate, LDH: lactate dehydrogenase.

Thiosemicarbazide derivatives **4** and **7** were also studied for their ability to interrupt the ATP hydrolysis reaction of topoisomerase IIA using the *E. coli* topoisomerase IV ATPase kit and *S. aureus* topoisomerase IV ATPase kit (INSPIRALIS, Norwich, UK, cat no. ATPECT001, ATPSAT001). Class IIA topoisomerases use energy from ATP hydrolysis for their proper action (supercoiling or decatenation). Some drugs, such as coumarins (e.g., novobiocin), inhibit hydrolysis by competing with ATP molecules for a binding site on the enzyme. The ATPase assay is based on the conversion of phosphoenolpyruvate (PEP) to pyruvate kinase (PK), coupled with the conversion of pyruvate to lactate by lactate dehydrogenase (LDH). This step requires NADH, which is oxidized to NAD+. NADH absorbs strongly at 340 nm but NAD+ does not; thus, the reduction of NADH over time can be measured by a decrease in absorbance (Scheme 1). The reaction was performed in 96-well, clear flat-bottomed plates and the protocol for gyrase and topoisomerase IV was the same. In brief, 1 U of the enzyme was incubated at 37 °C in a final volume of 100 μL containing 1× assay buffer, 800 μm PEP, 400 μm NADH, 1.5 μL PK/LDH, 10 nm linear pBR322 and the tested compound at a final concentration of 20 µM. Also, novobiocin at 20 µM was used as a control inhibitor. The mix was equilibrated for 10 min at 37 °C. The hydrolysis reaction was then initiated by the addition of ATP (2 mM) and the decrease in A_340_ was measured for 1 h at 5 min intervals. The decrease in OD_340_ over time was converted to ATP hydrolysis rate using an extinction coefficient of 6.22 mM^−1^ × cm^−1^ for NADH (and assuming 1 NADH molar equivalent to 1 ATP mol), and the percentage of inhibition compared to the untreated control was determined.

### 3.4. Antibacterial Activity

The in vitro antimicrobial activity of compounds **4** and **7** was evaluated using twelve clinical isolates of *S. aureus* received from the collection of the Department of Immunology and Infectious Biology, University of Łódź (described in [51]). All strains were kept frozen at −80 °C in Tryptic soy broth with 15% glycerol until testing. The minimum inhibitory concentration (MIC) was determined using two-fold dilutions of the tested compounds in Mueller–Hinton broth at a concentration range of 1–256 µg/mL in 96-well plates according to The European Committee on Antimicrobial Susceptibility Testing (EUCAST) guidelines (International Organization for Standardization, ISO 20776-1 (2006)). The final concentration of DMSO (compound diluent) was 1%, which did not influence bacterial growth. Bacteria were added at approximately 5 × 10^5^ CFU mL^−1^. The plates were incubated at 37 °C for 18 h and the optical density (OD_600_) was measured using a SpectraMax i3 Multi-Mode Platform (Molecular Devices, San Jose, CA, USA). Minimum bactericidal concentration (MBC), defined as the lowest concentration of a compound that resulted in a >99.9% reduction in CFU mL^−1^ of the initial inoculum, was determined following MIC evaluation by plating the contents of the first well that showed no visible growth of bacteria and the next two wells with higher concentrations of the compound onto Mueller–Hinton agar plates. Then, the plates were incubated at 37 °C for 18 h. Ampicillin, oxacillin, and nitrofurantoin were used as reference antimicrobial agents for both MIC and MBC determination. To evaluate the activity against *M. smegmatis* mc^2^ 155, nutrient broth (NB) (BD, Franklin Lakes, NJ, USA) was used, and the inoculum at a density of OD_600_ = 0.6–0.9 was used to prepare fresh cultures in 96-well plates starting at OD_600_ = 0.08, which then were incubated with the tested compounds for 48 h at 37 °C. Additionally, compound **7** was tested for its activity against *M. tuberculosis* H37Rv (American Type Culture Collection, ATCC 25618) cultured in Middlebrook 7H9 broth (BD, Franklin Lakes, NJ, USA) supplemented with 10% oleic acid, albumin, dextrose, and catalase (OADC) and incubated at 37 ˚C until the optical density of the inoculum reached OD_600_ = 0.080. Then, two-fold dilutions of the compounds were added, and the plate was incubated for 96 h at 37 °C. Determination of the MIC and MBC was conducted as described above. All evaluations were performed in triplicate.

### 3.5. ApoToxGlo Assay

To determine the type of cytotoxic activity induced by compounds **4** and **7**, the ApoTox-Glo™ Triplex assay (Promega, Madison, WI, USA) was performed. This test enables the simultaneous assessment of cell viability, drug cytotoxicity, and caspase activity. The viability of cells was determined fluorometrically using a GF-AFC (glycyl-phenylalanyl-aminofluorocoumarin) protease substrate conjugated to a fluorochrome. This substrate can penetrate the cell interior through the intact cell membrane, where it is cleaved by a protease active only in living cells. The protease cleavage of the substrate results in the emission of fluorescence, and its intensity is directly proportional to the number of living cells. The cytotoxic activity of the compounds was determined fluorometrically using a bis-AAF-R110 (bis-alanylalanyl-phenylalanyl-rhodamine 110) protease substrate coupled to a second fluorescent label. This substrate cannot penetrate the cell membrane; therefore, it is cleaved by a protease released from dead cells. The protease cleavage of the substrate results in the emission of fluorescence, and its intensity is directly proportional to the number of dead cells. The activity of caspases 3/7, which are markers of cells entering the apoptotic pathway, was measured using a third substrate containing the DEVD (Asp-Glu-Val-Asp tetrapeptide) caspase recognition sequence coupled to the luciferase substrate aminoluciferin. After cell lysis, caspases 3/7 cleaves the DEVD sequence and releases the luciferase substrate, resulting in luminescence emission, and its intensity is directly proportional to the caspase activity. The experiment was performed according to the manufacturer’s protocol. The HeLa cells (ATTC^®^ CCL-2™), cultured in Iscove’s Modified Dulbecco’s Medium Iscove’s Modified Dulbecco’s Medium (IMDM) medium supplemented with 10% FBS, were adjusted to a density of 7.5 × 10^3^ cells per well. The 96-well black plate with cells was incubated for 24 h at 37 °C and 10% CO_2_. After incubation, the growth medium was removed and 100 μL of fresh medium containing the tested compounds in the concentration range of 16–128 μg/mL was added (the DMSO content in the highest concentration of the compound did not exceed 1% and had no significant effect on cell growth). Cells grown without the addition of compounds were used as a positive control. Additionally, the activity of an antitumor compound (cisplatin) in the concentration range of 2–16 μg/mL was determined. After 24 h of incubation with the compounds, 20 µL of the viability/cytotoxicity assay reagent containing both substrates (GF-AFC and bis-AAF-R110) was added to each well. The plate was vigorously mixed and then incubated for 1 h at 37 °C and 10% CO_2_. The fluorescence was measured at Ex/Em = 400/505 nm (viability) and 485/520 nm (cytotoxicity) using the SpectraMax i3 Multi-Mode Platform (Molecular Devices, San Jose, CA, USA). For caspase activity determination, 100 µL of Caspase-Glo^®^ 3/7 reagent was added to all wells. The plate was incubated for 1 h at 37 °C and the luminescence was then measured. The results were presented as mean arithmetic values of relative fluorescence units (RFU) from two independent experiments.

### 3.6. Time-Lapse Microfluidic Microscopy

The DnaN-EGFP (JH01) strain used for TLMM experiments has been described in detail previously [56]. In short, the *dnaN* gene encoding the beta clamp of DNA polymerase III was replaced with the *dnaN-egfp* fusion gene in the native chromosomal locus.

TLMM was performed as previously described [52,56,57] using B04A plates and the CellASIC Onix flow-control system (Merck-Millipore, Burlington, MA, USA). Cells loaded into the observation chamber were exposed to fresh 7H9 + 10% OADC medium+ 0.05% Tween 80 for 5 h, followed by 7H9 + 10% OADC + 0.05% Tween 80 + inhibitor for 5 h, and then back again to fresh 7H9 + 10% OADC + 0.05% Tween 80 without antibiotic for 7 h under continuous pressure (1.5 psi) at 37 °C. Images were recorded at 10 min intervals using a Delta Vision Elite inverted microscope equipped with a 100× oil immersion objective and a CoolSnap HQ^2^ camera (Photometrics, Tucson, AZ, USA). The exposure conditions were as follows: for the FITC filter for EGFP illumination (excitation wavelength = 475/28 nm; emission wavelength = 525/48 nm): 80 ms and 50% intensity, and for the differential interference contrast (DIC): 50 ms and 5% intensity. All measurements were taken manually and analyzed with the ImageJ Fiji suite and R software platforms [58]. The *p*-value was determined by paired, two-sided, parametric Student’s *t*-tests with pooled SD. To avoid the generation of false assumptions in the case of non-normal distributions, statistical significance was confirmed with the non-parametric two-sided Wilcoxon test with minimum 0.995 confidence intervals.

## 4. Conclusions

The work presented previously [37,38,39] culminated in the identification of six thiosemicarbazide derivatives as initial prototypes of a novel class of bacterial DNA gyrase and topoisomerase IV inhibitors. Among them, 1-(indol-2-oyl)-4-(4-nitrophenyl)thiosemicarbazide (**4**) and 4-benzoyl-1-(indol-2-oyl)thiosemicarbazide (**7**) showed the highest inhibitory activities against topoisomerase IV (Topo IV) from *S. aureus*, with IC_50_ values of 14 μm. To expand these initial findings with further detail on their mode of action, the molecular docking approach combined with enzymatic studies and antimicrobial activity testing against clinical *S. aureus*, as well as *M. smegmatis* and *M. tuberculosis* strains, were conducted, followed by time-lapse microfluidic microscopy.

Docking studies revealed that two thiosemicarbazides with an indolamide core, **4** and **7**, bind to the ATP binding pocket of *S. aureus* ParE with much higher affinity than native kibdelomycin (a known inhibitor of GyrB/ParE subunits). Comparative analysis of KBD binding sites to the enzyme indicated that the indole nitrogen of **4** and **7** occupies an almost superimposable position with the pyrrole nitrogen of KBD and forms a hydrogen bond with the conserved Asp76 residue. Moreover, the binding of the indole moiety was stabilized by numerous hydrophobic interactions with surrounding residues, and most of them were identical to those in the crystal structure of the 43 kDa ParE–KBD complex. These results have been confirmed in vitro in enzymatic studies, which showed that the inhibition of Topo IV by **4** and **7** did not occur by stabilizing the cleavage complex as in the case of quinolones and fluoroquinolones. The evaluation of the level of ATP hydrolysis by Topo IV indicated that the tested compounds bind to the ParE subunit, competing for the binding site with the ATP molecule, which confirmed the earlier assumption from the docking studies. Compound **7** was found to be active against methicillin-resistant *S. aureus* strains (MRSA) and mycobacteria. Supplementary docking studies demonstrated the ability of compound **7** to bind to the ATPase region of *M. tuberculosis* GyrB; however, the in vitro analysis of *M. tuberculosis* gyrase activity in the presence of **7** did not show any inhibitory activity of this compound against the enzyme. Thus, it can be assumed that the anti-mycobacterial activity of **7** is due to its action on another molecular target in *Mycobacterium* cells. This microorganism does not have a typical Topoisomerase IV [59]; however, Jain and Nagaraya revealed the presence of a topoisomerase with decatenating activity in *M. smegmatis* that was different in its structure and origin, called Topo NM [60]. Perhaps the function of this enzyme is specifically impaired in the presence of **7**, or the function of some other important enzyme(s) is disrupted. These observations were further supported by the in vivo analysis using TLMM and a target-tagged fluorescent reporter strain of *M. smegmatis*. The real-time monitoring of replisome dynamics and cell elongation at the single-cell level enabled us to confirm our hypothesis (the novobiocin-like pattern of activity of the tested compounds on the replication process), but also revealed the existence of a putative additional mechanism of action resulting in a strong inhibition of bacterial growth. A thorough understanding of the basics of this mechanism requires further research. Thiosemicarbazide derivatives bearing indole moieties may be considered as novel inhibitors of the ATPase subunit of bacterial topoisomerases with dual antibacterial activity and open up the opportunity to be considered in the future as an alternative to aminocoumarin antibiotics and fluoroquinolones.

## Figures and Tables

**Table 1:** Scores of top poses of thiosemicarbazides **1**–**8** docked to proteins: PDB entry: 4URL (*S. aureus* 43 kDa ParE) and PDB entry: 4URM (*S. aureus* 24 kDa GyrB). * native ligand kibdelomycin.

	1	2	3	4	5	6	7	8	KBD *
*S. aureus* ParE									
(PDB ID: 4URL)	−29.0	−23.7	−38.0	−32.9	−33.1	−34.4	−37.0	−31.3	−26.8
*S. aureus* GyrB									
(PDB ID: 4URM)	−22.2	−23.5	−33.1	−33.0	−27.6	−27.4	−33.3	−29.4	−12.4

**Table 2:** ATPase activity of *S. aureus* topoisomerase IV treated with the tested compounds **4** and **7** (20 µM), demonstrated as a percentage of ATP hydrolysis inhibition compared to control (%) ± SEM from two independent experiments.

*S. aureus* Topoisomerase IV ATPase Assay			
Compound	4	7	Nov
ATP Hydrolysis Inhibition			
%	55.76 ± 1.59	49.35 ± 2.27	67.25 ± 10.50
	**	*	**

Nov: novobiocin (20 µM). * *p* < 0.05, ** *p* < 0.01 using one-way analysis of variance (ANOVA) with Tukey’s multiple comparison test.

**Table 3:** In vitro activity of **4** and **7** against clinical isolates of *S. aureus* expressed as the minimal inhibitory concentration (MIC) (µg/mL) and minimal bactericidal concentration (MBC) (µg/mL). AMP: ampicillin, OXA: oxacillin, NTF: nitrofurantoin.

	4		7		AMP		OXA		NTF	
Strain	MIC	MBC	MIC	MBC	MIC	MBC	MIC	MBC	MIC	MBC
**Nasopharynx Isolates**										
*S. aureus* C4	64	>256	16	128	>256	>256	0.4	0.4	16	16
*S. aureus* C7	64	>256	16	128	64	128	0.2	0.4	16	16
*S. aureus* C8	64	>256	16	128	>256	>256	1.5	1.5	16	16
*S. aureus* C19	64	>256	32	256	64	128	0.2	0.2	32	32
**Ulcer/Furuncle Isolates**										
*S. aureus* D12	64	>256	32	256	256	256	0.2	0.2	32	32
*S. aureus* F1	64	>256	16	128	128	128	0.8	0.8	32	32
*S. aureus* F7	64	>256	16	128	4	4	0.2	0.4	16	16
*S. aureus* F12	64	>256	8	64	64	128	0.2	0.2	16	32
**Bone Isolates**										
*S. aureus* D14	64	>256	32	>256	256	>256	0.2	0.2	32	32
*S. aureus* D15	64	>256	32	>256	>256	>256	64	128	32	32
*S. aureus* D17	64	>256	32	>256	>256	>256	64	128	32	32
*S. aureus* D20	64	>256	32	>256	128	256	0.4	0.4	16	32

**Table 4:** Antibacterial activity of **4** and **7** against *M. smegmatis* and *M. tuberculosis* (µg/mL). nd—not defined.

	4		7	
Strain	MIC	MBC	MIC	MBC
*M. smegmatis* mc^2^ 155	128	256	8	8
*M. tuberculosis* H37Rv	nd	nd	32	32

## Supplementary Materials

The following are available online at https://www.mdpi.com/article/10.3390/ijms22083881/s1, Video S1: TLMM analysis.avi.
